# Mapping Face Recognition Information Use across Cultures

**DOI:** 10.3389/fpsyg.2013.00034

**Published:** 2013-02-20

**Authors:** Sébastien Miellet, Luca Vizioli, Lingnan He, Xinyue Zhou, Roberto Caldara

**Affiliations:** ^1^Department of Psychology and Fribourg Center for Cognition, University of FribourgFribourg, Switzerland; ^2^School of Communication and Design, Sun Yat-Sen UniversityGuangzhou, China; ^3^Department of Psychology, Sun Yat-Sen UniversityGuangzhou, China

**Keywords:** face perception, culture, eye movements, gaze-contingent, expanding spotlight, extrafoveal processing

## Abstract

Face recognition is not rooted in a universal eye movement information-gathering strategy. Western observers favor a *local* facial feature sampling strategy, whereas Eastern observers prefer sampling face information from a *global*, central fixation strategy. Yet, the precise *qualitative* (the diagnostic) and *quantitative* (the amount) information underlying these cultural perceptual biases in face recognition remains undetermined. To this end, we monitored the eye movements of Western and Eastern observers during a face recognition task, with a novel gaze-contingent technique: the *Expanding Spotlight*. We used 2° Gaussian apertures centered on the observers’ fixations expanding dynamically at a rate of 1° every 25 ms at each fixation – the longer the fixation duration, the larger the aperture size. Identity-specific face information was only displayed within the Gaussian aperture; outside the aperture, an average face template was displayed to facilitate saccade planning. Thus, the *Expanding Spotlight* simultaneously maps out the facial information span at each fixation location. Data obtained with the *Expanding Spotlight* technique confirmed that Westerners extract more information from the eye region, whereas Easterners extract more information from the nose region. Interestingly, this quantitative difference was paired with a qualitative disparity. Retinal filters based on spatial-frequency decomposition built from the fixations maps revealed that Westerners used local high-spatial-frequency information sampling, covering all the features critical for effective face recognition (the eyes and the mouth). In contrast, Easterners achieved a similar result by using global low-spatial-frequency information from those facial features. Our data show that the face system flexibly engages into *local* or *global* eye movement strategies across cultures, by relying on distinct facial information span and culturally tuned spatially filtered information. Overall, our findings challenge the view of a unique putative process for face recognition.

## Introduction

Face-processing is a fundamental ability for social animals such as humans. Despite the vast amount of research on this topic, the exact nature and the specificity of the processes involved in this critical biological skill is a matter of ongoing debate. A potential reason for the discrepancies in the theoretical interpretations put forward by different authors might arise from a lack of consensus in the literature on the definition of the nature of the processes thought to be involved: namely the holistic, configural, and featural processing of faces. In a seminal review Maurer et al. (2002) stated that “Configural processing of faces can be divided into three types: (1) sensitivity to first-order relations – seeing a stimulus is a face because its features are arranged with two eyes above a nose, which is above a mouth; (2) holistic processing – gluing together the features into age stalt; and (3) sensitivity to second-order relations – perceiving the distances among features. However, there is no consensus about terminology…” More recently, McKone (2009) defined holistic or configural processing as “a special style of strong perceptual integration of information from across the entire internal region of a face” and acknowledges that “the exact nature of this style of computation is not understood.” Several authors in the present special issue make similar statements, agreeing on the lack of consensus in the definition of the concept: “Unfortunately, many studies provide only verbal descriptions of holistic processing and there is growing consensus that the concept is too loosely defined” (Richler et al., 2012) or “However, there is a lack of consensus and clarity in the literature regarding what is meant by holistic processing and how it is different from the part-based processing most commonly attributed to the perception of non-face objects” (Piepers and Robbins, 2012). We also believe that to date a *formal* – computational – unambiguous and accepted definition of any of the concepts above is still missing, creating confusion in the various interpretations of the many findings disseminated in the face literature. Stating that a particular experimental condition or group of observers is engaging in more holistic, configural, or featural processing for one particular task or another could be misleading if the exact nature of the process involved is not formally defined and all the alternative explanations are not properly discarded.

In order to tackle this issue and provide novel insights to this critical point, here we took advantage of the eye movements’ approach. Eye movements do not randomly sample the visual input space, but are effectively used by the brain to gather relevant information to adapt to the visual world. Therefore, they might reveal important information on the way humans process faces. In the past few years, we have designed a series of original experimental paradigms and developed a novel robust statistical approach (Caldara and Miellet, 2011) to isolate the information actively gathered by the eyes during face-processing. By isolating the visual information entering the face system, and the strategy used to sample this information, we can at least put minimal constraints on the algorithms enabling effective processing of faces. This approach might lead to a better understanding of the diagnostic information, the computation and nature of the representations involved in face-processing.

Numerous studies have used eye movement recordings to assess various aspects of face perception (for instance effect of memory: Althoff and Cohen, 1999; inverted faces: Williams and Henderson, 2007; familiar faces: Heisz and Shore, 2008). Some authors have claimed that the type of processing (i.e., hypothetically holistic or featural) impacts on oculomotor patterns (Bombari et al., 2009; Turati et al., 2010), sometimes assuming the involvement of the so-called holistic face-processing from fixation patterns (Chan and Ryan, 2012; Guo, 2012) or from behavioral performance during gaze-contingent paradigms (Van Belle et al., 2010a,b, 2011). Others have assumed that holistic face-processing can be independent of gaze behavior (de Heering et al., 2008).

Our recent studies showed differential oculomotor patterns during face recognition as a function of the culture of the observers, with a central fixation bias for the East Asian (EA) observers and an eye-mouth bias for the Western Caucasian (WC) observers (Blais et al., 2008). Such fixational biases seem to arise early in development, from 7 years old (Kelly et al., 2011b) and persist for inverted faces (Rodger et al., 2010), visually homogeneous objects (Kelly et al., 2010) and for second generation immigrants from an Eastern to a Western country (Kelly et al., 2011a). This cultural contrast in eye movement information sampling expands also to the categorization of facial expressions of emotions (Jack et al., 2009; Kelly et al., 2011a). However, the observed differences across cultures in face-processing do not generalize to visual search in complex natural scenes (Evans et al., 2009; Miellet et al., 2010).

By using gaze-contingent techniques, we have demonstrated that despite using diverse gaze scan paths, observers from both cultures rely on the same diagnostic features (i.e., the eyes and the mouth) to perform face recognition and to reach comparable levels of performance (Caldara et al., 2010; Miellet et al., 2012). Importantly, we have developed a novel gaze-contingent technique – the *i*Hybrid – and recently reported that WC observers identify famous faces on the basis of both *foveally* (local) and *extrafoveally* (global) sampled information, depending on the first fixation landing position (Miellet et al., 2011). All observers used both strategies, often to recover the very same identity. Altogether these findings allow us to draw the following conclusions. Firstly, all human observers can use different visual strategies to recognize faces. Secondly, culture impacts upon the preferred information sampling strategy during face recognition. EA observers are more inclined to sample facial diagnostic information from extrafoveal vision, whereas WC observers preferentially use foveal information. These observations suggest that the facial information used to accurately individuate conspecifics is invariant across human beings, but the strategies used to extract this information are likely to be flexible and might be modulated by culture. Thirdly, these results confirm that the coupling between fixated and processed information is not perfect (concepts of overt vs. covert attention, Posner, 1980). Hence, it would be misleading to infer hypothetical holistic/configural vs. featural processing/representations based only on fixation locations with static images. In contrast, gaze-contingent techniques warrant a fine and on-line control of the available information, therefore allowing stronger conclusions in terms of potential information use. Finally, if the same diagnostic features are sampled from different fixation locations across cultures, one would assume that the visual input received by the face system is different. Yet, the precise *qualitative* (i.e., the diagnostic) and *quantitative* (i.e., the amount) information underlying these cultural perceptual biases in face recognition remains undetermined.

In order to further clarify how the face system achieves face identification across cultures, in the present study we introduced a novel gaze-contingent technique: the *Expanding Spotlight*. In previous studies we used a moving window (*Spotlight*, Caldara et al., 2010) or a moving mask (*Blindspot*, Miellet et al., 2012) and parametrically manipulated the size of the window/mask (0° = natural vision, 2°, 5°, and 8° of visual angle) in order to obtain a fine assessment of the effect of the gaze-contingent manipulation on oculomotor scan paths, information use, and performance. However, these manipulations did not allow precise measurement of the quantity and quality of information – information span – sampled at every facial location, as for a given condition the *Spotlight* or *Blindspot* size was constant regardless of the fixation location. In addition, despite having had clear arguments for determining *a priori* the aperture sizes of our masks, one could not completely exclude a potential bias arising from the choice of these parameters.

**Figure 1:**
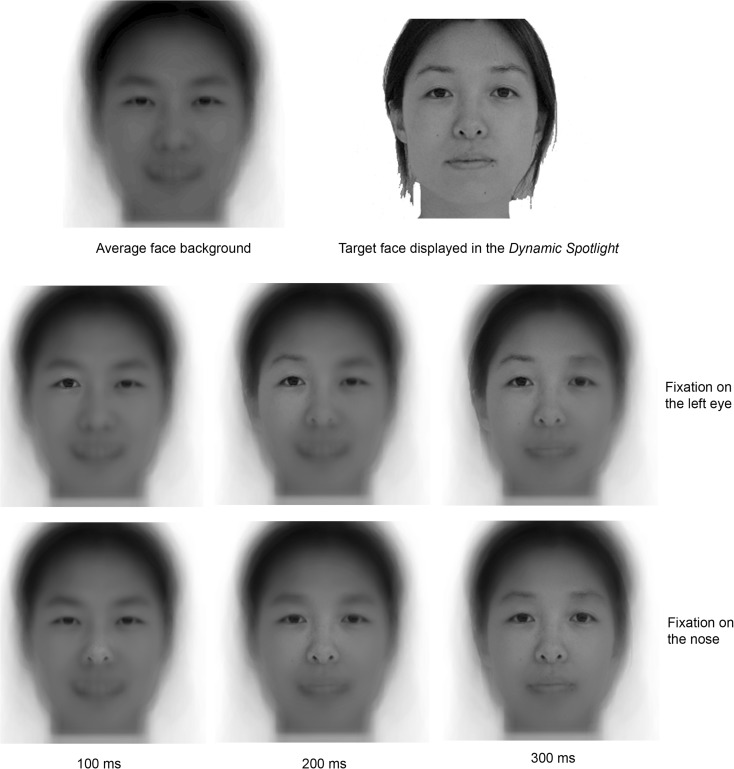
**Illustration of the *Expanding Spotlight* technique**. The first row shows on the left the average face presented outside the gaze-contingent foveal Gaussian aperture; and on the right an example of a target face displayed at the fixation location. The second and third rows illustrate the expansion of the *Spotlight* across time; they show examples of stimulus display after 100, 200, and 300 ms fixation (first, second, and third column respectively). The second line illustrates the *Expanding Spotlight* with a fixation on the left (on the screen) eye; the third line illustrates the *Expanding Spotlight* with a fixation on the center of the face. Note that there is no direct relationship between the Gaussian aperture size and the availability of information in the full spatial-frequency spectrum. This is due to the overall Gaussian shape.

To overcome these limitations, in the present study the facial information corresponding to the target identity was available only inside a Gaussian aperture dynamically centered on the participant’s fixation location. The gaze-contingent Gaussian aperture expanded with time (1° every 25 ms). The longer was the fixation duration, the larger the *Spotlight* aperture size became. The *Spotlight* aperture was contracted to 2° (foveal region) at each new fixation. We replaced information outside central vision with an average face template, to allow saccade programming and natural fixation sequences, although not providing useful information for the recognition task. Figure 1 represents the expansion of the *Spotlight* across time. Our main assumption is that, with the *Expanding Spotlight*, observers would maintain a fixation to a given location until they obtain sufficient foveal and extrafoveal information from this location to solve the task at hand. The use of an average face template provides them with the information necessary to program the next saccade, therefore weakening the view of maintaining fixation for this purpose only. In addition, human vision is governed by a coarse-to-fine (e.g., Winston et al., 2003) or broad-to-fine (van Rijsbergen and Schyns, 2009) processing over time, which again weakens the view suggesting that fixations in the *Expanding Spotlight* technique would be maintained for the purpose of saccade planning. Of course, the *Expanding Spotlight* involves a direct relationship between the fixation duration and the spatial extent of available information, which in some circumstances could inflate the importance of extrafoveal information revealed when the observer is finely analyzing foveated information. However, such a bias will be uncovered by the reconstructed information span images based on a foveated retinal filter. Therefore, the *Expanding Spotlight* is an active gaze-driven technique that allows precise isolation of the information span at each fixation.

An illustration movie can be found at http://perso.unifr.ch/roberto.caldara/movies/exp_spot.mov. It is worth noting that the *Spotlight* expansion is slowed down in this example in order to make the technique easy to grasp.

Here, we used the *Expanding Spotlight* with WC and EA observers performing an old-new face recognition task with WC and EA faces. Our rationale was that the participants would maintain their fixations for the time necessary to effectively encode the diagnostic face features of interest. Our main goal was to map precisely the quantitative and qualitative information necessary for face recognition across culture. Specifically, with the *Expanding Spotlight*, it is possible to establish precisely the information span for every single fixation/feature entering in the face system. Finally, this information was convoluted with a retinal filter to reconstruct a precise picture of the visual input used by observers from different cultures to achieve face recognition.

## Materials and Methods

### Participants

Fifteen Western Caucasian (11 females) and 15 EA (nine females) young adults (mean age 24.32 and 22.45 years respectively) participated in this study. The Western Caucasian participants were students at the University of Glasgow, UK and the EA participants were students at the Sun Yat-Sen University, Guangzhou, China. All participants had normal or corrected vision and were paid £6 or equivalent per hour for their participation. All participants gave written informed consent and the protocol was approved by the ethical committees of the Department of Psychology of the University of Glasgow and the ethical committee of the Department of Psychology of the University of Sun Yat-Sen University.

### Materials

Stimuli were obtained from the KDEF (Lundqvist et al., 1998) and AFID (Bang et al., 2001) databases and consisted of 56 EA and 56 Western Caucasian identities containing equal numbers of males and females. The images were 382 × 390 pixels in size, subtending 15.6° of visual angle vertically and 15.3° of visual angle horizontally, which represents the size of a real face (approximately 19 cm in height). Faces from the original databases were aligned by the authors on the eye and mouth positions; the images were rescaled to match those facial features position and normalized for luminance. Images were viewed at a distance of 70 cm, reflecting a natural distance during human interaction (Hall, 1966). All images were cropped around the face to remove clothing and were devoid of distinctive features (scarf, jewelry, facial hair, etc.). Faces were presented on a 800 × 600 pixel gray background displayed on a Dell P1130 21″ CRT monitor with a refresh rate of 170 Hz.

### Apparatus

Eye movements were recorded at a sampling rate of 1000 Hz with the SR Research Desktop-Mount EyeLink 2K eyetracker (with a chin/forehead rest), which has an average gaze position error of about 0.25°, a spatial resolution of 0.01° and a linear output over the range of the monitor used. Only the dominant eye was tracked, although viewing was binocular. The experiment was implemented in Matlab (R2009b, The MathWorks, Natick, MA, USA), using the Psychophysics (PTB-3) and EyeLink Toolbox extensions (Brainard, 1997; Cornelissen et al., 2002). Calibrations of eye fixations were conducted at the beginning of the experiment using a nine-point fixation procedure as implemented in the EyeLink API (see EyeLink Manual) and using Matlab software. Calibrations were then validated with the EyeLink software and repeated when necessary until the optimal calibration criterion was reached. At the beginning of each trial, participants were instructed to fixate a dot at the center of the screen to perform a drift correction. If the drift correction was more than 1°, a new calibration was launched to insure an optimal recording quality. The eyetracker, software and settings used in Glasgow and Sun Yat-Sen universities were identical.

### Expanding spotlight

The *Expanding Spotlight* had a zero alpha value at the center. The alpha value is the value of the alpha channel we used to create the Gaussian apertures combined with an image as background to create the appearance of partial transparency. This value increased with distance from center of gaze according to a Gaussian function and reached one (complete opacity) at the border of the aperture. We used 2° Gaussian apertures centered on the observers’ fixations expanding dynamically at a rate of 1° every 25 ms at each (novel) fixation, without expansion limit constraints. We chose this expansion rate for two reasons. Firstly, the expansion rate is sufficiently *fast* to keep the fixation durations in the average range observed in natural vision during face-processing. Hence, with this expansion rate, the *Spotlight* reaches the size of 14°of visual angle in 300 ms. A 14° Gaussian aperture allows the participant to process eyes and mouth from a fixation on the center of the face (note that smaller apertures, 8° in Caldara et al., 2010, produced fixation patterns similar to those observed in the baseline/natural vision condition). Secondly, this expansion rate is sufficiently *slow* to give enough sensitivity to highlight difference between local and global sampling strategies. For instance, at least 100 ms are necessary for the *Spotlight* to expand from a Gaussian covering the size of an eye (between 2° and 8° – cf. Figure 1, middle row, first and second faces) to a Gaussian covering both eyes and mouth from a central fixation (roughly 14° – cf. Figure 1, bottom row, third face). The image outside the *Expanding Spotlight* was an average face template composed from all the stimuli used in the experiment, allowing the observers to program natural saccades. Importantly, the face template does not provide any useful information for the recognition task. The display contingent to gaze position updating required 11 ms on average (between 8 and 14 ms), eliminating any impression of flickering for the observers.

### Procedure

All the participants started with a training session in order to familiarize them with the gaze-contingent display. Then they were informed that they would be presented with a series of faces to learn and subsequently recognize. In each of the eight experimental blocks, the observers were instructed to learn seven face identities randomly displaying either neutral, happy, or disgust expressions. After a 30 s pause, a series of 14 faces (seven faces from the learning phase – seven new faces) were presented and observers were instructed to indicate as quickly and as accurately as possible whether each face had been presented in the learning phase or not by pressing keys on the keyboard with the index of either their left or right hand. Response times and accuracy were collected and analyzed for the purpose of the present experiment. Response buttons were counterbalanced across participants. The emotional expression of the faces was changed between the learning and the recognition stage to avoid trivial image-matching strategies.

Each trial started with the presentation of a central fixation cross. Then four crosses were presented, one in the middle of each of the four quadrants of the computer screen. These crosses allowed the experimenter to check that the calibration was still accurate. In this way, we validated the calibration between each trial. A final central fixation cross served as a drift correction, followed by a face presentation. Faces were presented for 5 s in the learning phase and until the observer’s response in the recognition phase. To prevent anticipatory strategies, images were presented at random locations on the computer screen. Each trial was subsequently followed by the six fixation crosses which preceded the next face stimulus.

### Data analyses

**Figure A1:**
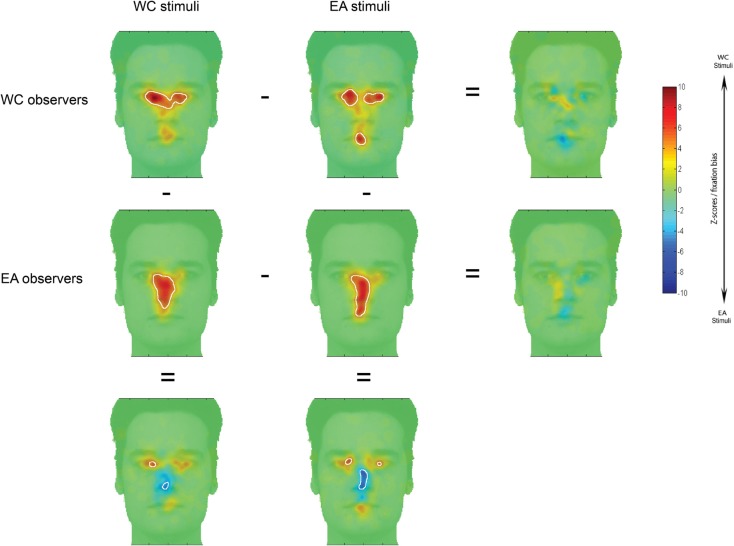
**Fixation maps based on the fixation durations for each culture of the observer and each race of the stimuli**. Subtracting fixation maps across culture of the observer or race of the stimuli resulted in difference maps. On observers/stimuli maps shown here, white contours surround regions of significantly longer fixation durations than were observed for other areas; on the difference maps, white contours indicated regions of significant differences between the observers/stimuli maps (i.e., fixation bias).

Behavioral performance was measured by the percentages of correct recognition and the reaction time. Trials further than two standard-deviations from the participant’s average duration were discarded (3% of the trials). Saccades and fixations were determined using a custom algorithm using the same filter parameters as the EyeLink software (saccade velocity threshold = 30°/s; saccade acceleration threshold = 4000°/s^2^) and merging fixations close spatially and temporally (<20 ms, <0.3°). Fixation distribution maps were extracted individually for each observer. Previous studies did not reveal any impact of the task (learning vs. recognition), correct vs. incorrect trials or race of the face stimulus (WC vs. EA) on the statistical fixation maps (Blais et al., 2008; Caldara et al., 2010; Kelly et al., 2010). Here, we analyzed the correct recognition trials (65.1 and 65.8% of the trials for EA and WC observers respectively) and we collapsed data for the EA and WC face stimuli given the lack of effect of the race of the face stimuli (see Figure A1 in Appendix). We computed various variables describing the general oculomotor behavior in order to insure that EA and WC information sampling strategies are comparable (number of fixations per trial, average total fixation duration per trial, average single fixation duration, scan path length per trial, and average saccade length). The statistical fixation maps were computed with the *i*Map toolbox (version 2.1, Caldara and Miellet, 2011). *i*Map establishes significance using a robust statistical approach correcting for multiple comparisons in the fixation map space, by applying a one-tailed Pixel test (Chauvin et al., 2005; *Z*_crit_ for the present search space >4.07; *p* < 0.05) for the group fixation maps and a two-tailed Pixel test (*Z*_crit_|4.25|; *p* < 0.05) on the differential fixation maps. Finally, for each condition we extracted the average *Z*-score values for each observer individually, within the regions showing significance in the differential fixation maps. Cohen’s *d* effect sizes (Cohen, 1988) of culture were calculated on the average *Z*-scores for each region showing significance.

## Results

We observed similar reaction times and performance for EA and WC observers [see Table 1, both *t*(28) < 1]. Likewise, the global eye-tracking measures did not reveal any significant difference between both groups of observers [See Table 2, all *t*(28) < 1.1].

**Table 1:** **Performance (*d*′) and reaction times (RT) according to the culture of the observer**.

	WC observers	EA observers
RT (ms)	223 (11)	224 (22)
*d*′	0.86 (0.12)	0.91 (0.11)

*The *d*′ is calculated as *Z*(hit rate)−*Z*(false alarm rate). The standard errors from the mean are reported in brackets*.

**Table 2:** **Global eye-tracking measures to the culture of the observer: average number of fixations per trial, average total fixation duration per trial (in ms), average single fixation duration (in ms), scan path length per trial (in degrees on visual angle), and average saccade length (in degrees on visual angle)**.

	WC observers	EA observers
Number of fixations per trial	3.81 (0.48)	4.63 (0.64)
Total fixation duration per trial (ms)	1321 (160)	1606 (216)
Fixation duration (ms)	334 (13)	344 (7)
Path length (degree)	8.36 (0.93)	9.16 (1.02)
Saccade length (degree)	1.93 (0.07)	1.96 (0.08)

*The standard errors from the mean are reported in brackets*.

**Figure 2:**
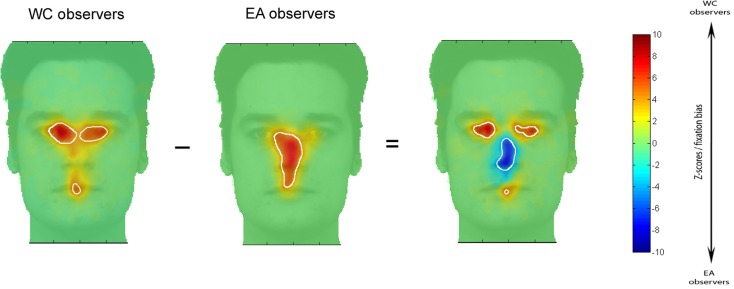
**Fixation maps based on the fixation durations for each culture of the observer**. Subtracting the fixation map for EA observers from the fixation map for WC observers resulted in a difference map. On the EA and WC maps shown here, white contours surround regions of significantly longer fixation durations than were observed for other areas; on the difference map, white contours indicated regions of significant differences between the EA and WC maps (i.e., fixation bias).

Figure 2 shows fixation maps and the regions significantly fixated above chance level according to *i*Map (white contours) for EA and WC observers during face recognition. The difference maps reveal the well-established central bias for Easterners (in blue in the difference map) and eye-mouth bias for Westerners (in red in the difference map) in the Natural vision condition.

In order to determine the magnitude of the fixation biases across cultures, we extracted, for each observer, the average of the *Z*-scored fixation durations within the areas showing significant differences in the differential fixation maps for WC-EA (Figure 2). Then we carried out *t*-tests on the averaged *Z*-score values with Culture of the observer as a between-subjects factor. WC observers spent significantly longer fixating the eyes-mouth region than EA observers (286 and 106 ms. respectively) as revealed by a two tailed *t*-tests [*t*(28) = 3.09, *p* < 0.005]. In contrast, EA observers fixated longer on the center of the face than WC observers [368 and 100 respectively; *t*(28) = 3.64, *p* < 0.001]. Cultural fixation biases on facial features were reliable and robust, as highlighted by the large magnitude of Cohen’s *d* effect size values for the significant effects: 0.92 for the effect of culture in the eyes-mouth region and 1.06 for the effect in the center of the face. The effect sizes were calculated on data-driven areas showing statistical significant contrasts (and not pre-defined ROIs). However, the same data set was used to both determine the significant areas with *i*Map and compute the effect sizes from these significant areas (for selection and selective analysis). Henceforth, the non-independent selective analysis might distort descriptive statistics (see Kriegeskorte et al., 2009; example 2). Therefore, to validate these results we reiteratively computed the effect sizes with a bootstrapping procedure (500 resamples) with independent random split-data analyses. Thus, we used half of the participants of each group for the selection analysis (determining the significant areas according to *i*Map) and the other half for the selective analysis (calculation of the effect sizes within the significant regions). This analysis revealed a range of effect sizes that encompasses those computed from the full data set (median = 0.61, SD = 0.73 for the effect of culture in the eyes-mouth region and median = 1.31, SD = 0.67 for the effect in the center of the face), corroborating the conventional analysis.

**Figure 3:**
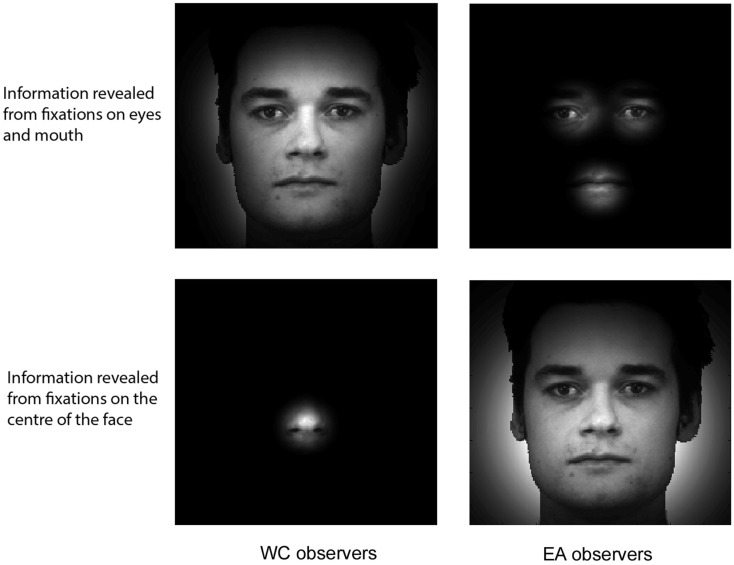
**Reconstructed available information as a function of facial fixation locations and the culture of the observer**. The size of the Gaussian aperture is determined by the expansion rate of the *Expanding Spotlight* and the average fixation duration in the corresponding significant area.

The *Expanding Spotlight* allows us to represent the quantity of information available in the stimulus as a function of the fixation location and duration. Figure 3 shows the available information, which was obtained by centering a Gaussian aperture on the local maximum of each significant area for each group of observer. The aperture size was function of the expansion rate and the average fixation duration per trial in the significant area.

**Figure 4:**
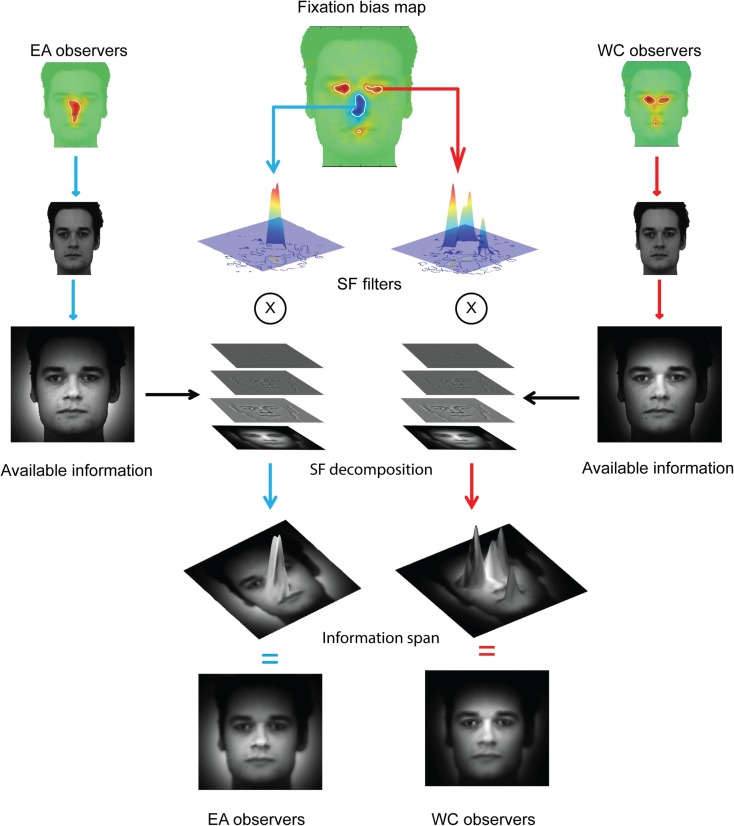
**Isolating the information span**. The reconstructed available information (see Figure 3) is decomposed in four spatial-frequency (SF) bands covering the full spatial-frequency spectrum. The SF bands are then convoluted on the cultural fixation biases extracted from the differential fixation map (see Figure 2). See Figure 5 for a fine-grained illustration of the information span.

**Figure 5:**
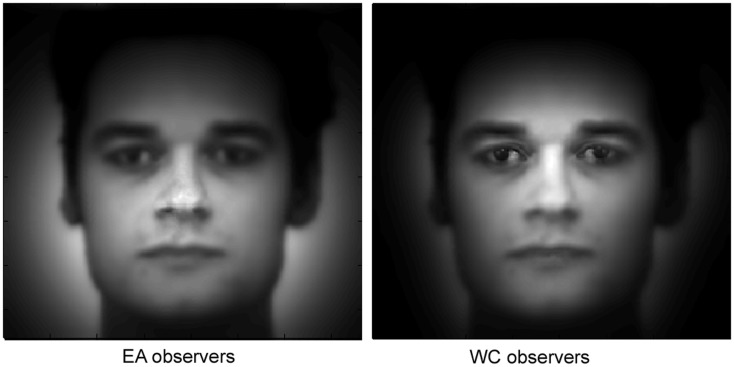
**Information span for the global (EA) and local (WC) strategies**.

We also reconstructed the visual information available to the face system. To this aim, we used a retinal filter based on spatial frequencies decomposition (see Miellet et al., 2011) and convoluted the information available in the stimulus with the fixation bias of each group of observers. We reconstructed the sampled information by decomposing the total information revealed by each group of observers (see Figure 3) into a four-level Laplacian pyramid (Simoncelli and Freeman, 1995). We then filtered each spatial-frequency band by applying a WC- or EA-retinal filter, which were built from the significant statistical contrasts between groups on the fixation maps (see Figure 2). Figure 4 shows the method to compute the reconstructed sampled information. The resulting information span is shown in Figure 5.

## Discussion

The aim of this study was to isolate the precise *quantitative* and *qualitative* facial information intake during face recognition in Western Caucasian (WC) and EA observers. In line with our previous findings (e.g., Blais et al., 2008; Caldara et al., 2010; Miellet et al., 2012), WC and EA observers deployed distinct information sampling strategies to encode and recognize faces, reaching comparable recognition performance. WC observers deployed *local* fixations directed toward the eyes and the mouth. EA observers deployed a *global* information sampling strategy typified by a central fixation pattern. Yet, reaction times, accuracy measures (*d*′) and global eye movement indices (i.e., number of fixations, total fixation duration, single fixation duration, path, and saccade lengths) did not differ across cultures.

The novelty here relies on the original methodological approach: the *Expanding Spotlight*. The results on the global eye movement indices were compatible with previous data obtained in natural viewing conditions (Blais et al., 2008; Miellet et al., 2012 and the other cultural studies) or with the *i*Hybrid technique (Miellet et al., 2011), attesting for the ecological validity of the technique. Yet, in all of our previous studies we reported *qualitative* effects. A significant contrast with *i*Map (i.e., region delimited by a white border) does not precisely inform on the *quantity* of foveal and extrafoveal information sampled from this region. For the first time, the *Expanding Spotlight* isolated the facial information span used by observers from different cultures, by reconstructing this information with a retinal filter based on spatial-frequency decomposition on the fixation maps. The reconstructed information span images revealed that this visual bias was related to a spatial-frequency tuning. These novel findings clearly show that this information is original and complementary to the significant fixation contrasts revealed by *i*Map and, crucially, cannot be simply revealed from the regions fixated above chance level in the fixation maps. To the best of our knowledge, this is the first time that the information span for faces (across cultures) is revealed.

Our data directly challenge the idea of a unique process and representation format dedicated to face-processing. An exclusive strict holistic position of face-processing, according to which all the facial information has identical importance, could not account for our observations. Observers from different cultures used different scan paths to recognize faces, which critically led to the use of different visual inputs (as shown by the reconstructed retinal filter images). As a result, it becomes difficult to extrapolate that observers from different cultures use the same mandatory holistic processing of faces. Such a strict holistic view would need to explain how a unique computation carried on different visual inputs would lead to identical (holistic) representations. Our data suggest instead a cultural tuning in the facial information space, which most probably rely on distinct cultural representations.

Obviously, we do not deny the capability of human beings to bind visual information into a global percept; and that the familiarity of the configuration might help binding information (see for instance Kessler and Miellet, 2012). However, we feel that a strictly holistic account of face-processing raises at minimum a few major caveats. Firstly, the concept of holistic processing is underspecified, making it easy to account for a large number of results. To the best of our knowledge, there is not a commonly acknowledged definition of holistic processing for faces in the literature, specifying the precise information related to this process. Hence, researchers might refer to different processes when they account results in terms of holistic processing. Moreover, the compatibility of a pattern of results with a theoretical holistic account does not guarantee that the actual processing taking place in the experimental tasks is holistic in nature. In its most comprehensive meaning, we could consider that holistic refers to the binding and integration of diagnostic information into a coherent percept, in which some features (i.e., the eyes) are more diagnostic than others. In our view, then the very nature of holistic processing of faces should be clarified by using a formal approach, mapping the diagnosticity of every facial feature, weighting interactions across features, and ascertaining the modulations of this multidimensional space as a function of task constraints. Having such a formal model would also allow us to formulate predictions and test the validity of the model at the experimental level. In this model, eye movements could also inform how information is cumulated across fixations and how face representations are effectively built. However, based on current knowledge of this theoretical question, and the lack of a formal model, we think that the gap between the so-called holistic processing of faces and the information binding routinely engaged in natural vision is unclear. Secondly, and more specifically concerning eye movement research, a common theoretical shortcut has been put forward between extrafoveal information sampling and holistic processing/representations. For instance Hsiao and Cottrell (2008) assumed that a central fixation pattern supports holistic processing for faces (the sampled information considered to be the eyes). It is important to note that in this study the authors used the very same stimuli for the learning and recognition phases, potentially allowing observers to use low-level image-matching strategies instead of genuine face recognition processes. Moreover, Hsiao and Cottrell used only two possible stimulus locations (above and below the central fixation point), which did not prevent the observers from using anticipatory oculomotor strategies. In many other studies, it is assumed that because the observers do not directly fixate the diagnostic information, the processing is holistic (e.g., Guo, 2012). We think that this is not necessarily the case; such fixation patterns only tell us that the diagnostic information might have been sampled extrafoveally. Moreover, in one of our studies (Kelly et al., 2010) EA observers deployed central fixations to human faces, sheep faces, and Greebles, questioning the specificity of the holistic, central/global fixation strategies for faces. As a consequence, we do not think that we can account for those processes with such straightforward binary distinction (i.e., extrafoveal fixation = holistic processing vs. foveal fixation = featural processing). We thus believe that natural eye movement recordings do not provide direct evidence on the nature of the representations, and can only isolate oculomotor strategies.

Yet, gaze-contingent techniques are more informative as they control for the available information and offer some insights on the computations devoted to information use. On this issue, gaze-contingent techniques artificially control and manipulate the available information, by constraining it to the fixated location. Therefore, at first sight one could assume that findings gathered with these techniques might be difficult to relate to natural vision, and are instead tapping into unnatural visual processes. Yet, this view is invalidated by experimental and theoretical evidence. More concretely, here we used an average face template (with normalized eyes and mouth positions) to cover the information presented outside the *Expanding Spotlight*. A full-face was thus always available to the observers, an experimental manipulation that enabled them to program the next saccade and precisely target a landing location. Moreover, we intentionally used a Gaussian aperture with a progressive opacity to avoid a highly contrasted hard border, which would have increased visual saliency and inevitably attract attention between the background (average face template) and the *Expanding Spotlight* (stimulus). Critically, and more objectively, our data are fully compatible with previous results obtained in natural viewing. Firstly, fixation durations are in the same range as non-gaze-contingent eye-tracking studies on face-processing in natural vision (Henderson et al., 2005; Hsiao and Cottrell, 2008). Secondly, the average number of fixations is similar to those gathered from a study using the same old/new task and a natural vision baseline (Miellet et al., 2012). Thirdly, we previously showed that ambiguous hybrid face representations reconstructed from the eye movement scan paths of gaze-contingent data could successfully predict the facial identify perceived by an independent group of observers (Miellet et al., 2011). Finally, the present eye movement patterns are perfectly in line with the cultural bias observed for face-processing in numerous studies with natural vision paradigms (Blais et al., 2008; Kelly et al., 2010, 2011a,b; Rodger et al., 2010; Miellet et al., 2012). Altogether, these experimental and theoretical arguments converge and convincingly demonstrate that gaze-contingent techniques can be a valuable and unique tool to isolate and finely understand information processing in natural vision.

Despite these considerations, it is important to mention that gaze-contingent techniques do not directly uncover representation formats. For instance, Van Belle and colleagues (Van Belle et al., 2010a,b, 2011) make the direct, although implicit assumption that extrafoveal information sampling (promoted by a gaze-contingent central mask) is equivalent to holistic processing, whereas foveal sampling (promoted via a gaze-contingent central window) corresponds to featural processing. We argue that foveal information sampling could potentially result in either featural or holistic representations. For example, motor information from saccades on facial features might aid their integration into a spatial/configural representation, resulting in a global representation. Conversely, extrafoveal information can be used to build featural information, detailed enough to produce effective recognition. To sum up, even if gaze-contingent techniques allow precisely isolating and characterizing the information span, it is still problematical to unambiguously conclude on the nature of the face representations.

Given those considerations, an important question we should ask ourselves is whether the label “holistic” is conceptually appropriate for face-processing in the context of the eye movement research. We genuinely believe that this concept is not critical in this framework as it does not provide insightful and objective explanations. We suggest, instead to remain closer to what the eye movement data show and to shift the focus on crucial questions, such as for instance: what factors are modulating the eye movement strategies, which information is used for a given task and under what specific visual constraints, how this information is bound to form a percept that taps into the face representation, how information is integrated across saccades? These questions and many important others remain unanswered and we believe that eye movement data will provide unique and important information to address them. To date, our eye movement data have revealed the existence of diverse strategies dedicated to face-processing, strategies that adjust to the task at hand, the visual constraints, and the culture of the observer. These observations challenge the view of a unique process dedicated to face-processing, but future studies are necessary to understand the roots and rules driving such (cultural) diversity in the way humans sample visual information.

To conclude, we favor a view according to which eye movements are flexibly used to elaborate face representations by gathering information through diverse strategies. With gaze-contingent techniques it is also possible to precisely map out facial information span, an original and rich advantage compared to other approaches in visual cognition. This is consistent with the idea that the type of processing might vary with distance (McKone, 2009), familiarity with the face, task constraints, culture of the observer, etc. Even if faces are a special class of visual stimuli (through frequency of exposure, social relevance, and/or adaptive advantage), their processing is still governed by the visual system. The present data clearly show that Western Caucasian observers favor *local* eye movement strategies to process faces, whereas EA observers favor a *global* strategy. Both groups rely on distinct facial information span and culturally tuned spatially filtered information. These observations suggest that human vision is modulated by culture and continuously using *local* and *global* information to process human faces effectively, but most probably also to decode the visual world.

## Conflict of Interest Statement

The authors declare that the research was conducted in the absence of any commercial or financial relationships that could be construed as a potential conflict of interest.

## Supplementary Material

The Supplementary Material for this article can be found online at http://www.frontiersin.org/Perception_Science/10.3389/fpsyg.2013.00034/abstract
